# Testing the Impact of the #chatsafe Intervention on Young People’s Ability to Communicate Safely About Suicide on Social Media: Protocol for a Randomized Controlled Trial

**DOI:** 10.2196/44300

**Published:** 2023-02-17

**Authors:** Jo Robinson, Louise La Sala, Charlie Cooper, Matthew Spittal, Simon Rice, Michelle Lamblin, Ellie Brown, Hayley Nolan, Rikki Battersby-Coulter, Gowri Rajaram, Pinar Thorn, Jane Pirkis, Summer May-Finlay, Vincent Silenzio, Jaelea Skehan, Karolina Krysinska, India Bellairs-Walsh

**Affiliations:** 1 Orygen Parkville Australia; 2 Centre for Youth Mental Health University of Melbourne Parkville Australia; 3 Centre for Mental Health School of Population and Global Health University of Melbourne Parkville Australia; 4 School of Health and Society Faculty of the Arts, Social Sciences and Humanities University of Wollongong Wollongong Australia; 5 Department of Urban-Global Public Health Rutgers School of Public Health Rutgers University Piscataway, NJ United States; 6 Everymind Newcastle Australia; 7 College of Health, Medicine and Wellbeing University of Newcastle Newcastle Australia

**Keywords:** suicide, young people, social media, intervention, sexual health, randomized-controlled trial

## Abstract

**Background:**

Suicide is the leading cause of death among Australians. One commonly cited explanation is the impact of social media, in particular, the ways in which young people use social media to communicate about their own experiences and their exposure to suicide-related content posted by others. Guidelines designed to assist mainstream media to safely report about suicide are widespread. Until recently, no guidelines existed that targeted social media or young people. In response, we developed the #chatsafe guidelines and a supporting social media campaign, which together make up the #chatsafe intervention. The intervention was tested in a pilot study with positive results. However, the study was limited by the lack of a control group.

**Objective:**

The aim of this study is to assess the impact of the #chatsafe social media intervention on young people’s safety and confidence when communicating on the web about suicide.

**Methods:**

The study employs a pragmatic, parallel, superiority randomized controlled design. It will be conducted in accordance with the Consolidated Standards of Reporting Trials statement over 18 months. Participants will be 400 young people aged 16-25 years (200 per arm). Participants will be recruited via social media advertising and assessed at 3 time points: time 1—baseline; time 2—8-week postintervention commencement; and time 3—4-week postintervention. They will be asked to complete a weekly survey to monitor safety and evaluate each piece of social media content. The intervention comprises an 8-week social media campaign including social media posts shared on public Instagram profiles. The intervention group will receive the #chatsafe suicide prevention content and the control group will receive sexual health content. Both groups will receive 24 pieces of content delivered to their mobile phones via text message. The primary outcome is safety when communicating on the web about suicide, as measured via the purpose-designed #chatsafe online safety questionnaire. Additional outcomes include willingness to intervene against suicide, internet self-efficacy, safety, and acceptability.

**Results:**

The study was funded in November 2020, approved by the University of Melbourne Human Research Ethics Committee on October 7, 2022, and prospectively registered with the Australian New Zealand Clinical Trials registry. Trial recruitment began in November 2022 and study completion is anticipated by June 2024.

**Conclusions:**

This will be the first randomized controlled trial internationally to test the impact of a social media intervention designed to equip young people to communicate safely on the web about suicide. Given the rising rates of youth suicide in Australia and the acceptability of social media among young people, incorporating social media–based interventions into the suicide prevention landscape is an obvious next step. This intervention, if effective, could also be extended internationally, thereby improving web-based safety for young people not just in Australia but globally.

**Trial Registration:**

Australian New Zealand Clinical Trials Registry ACTRN12622001397707; https://anzctr.org.au/Trial/Registration/TrialReview.aspx?id=384318

**International Registered Report Identifier (IRRID):**

DERR1-10.2196/44300

## Introduction

### Background

Suicide is the leading cause of death among Australians younger than 25 years, and rates have been increasing over the past 10 years [1]. The pathways that lead a young person to feel suicidal are complex and diverse; however, 1 commonly cited explanation for the recent rise in rates of youth suicide is the impact of social media [2].

Young Australians spend over 3 hours per day on social media, and this sometimes includes communicating with others about their own experiences of suicide and being exposed to suicide-related content posted by others [2-4]. While there are many potential benefits to communicating on the internet about suicide, such as the ability to seek and provide support from others in an accessible and nonstigmatizing manner [3,5], the potential for harm also exists. For example, there are concerns that certain types of content (eg, graphic information or images) may cause distress, lead to imitative suicidal behavior, and have been thought to contribute to suicide clusters [6]. In addition, young people may be exposed to expressions of suicide risk posted by others but feel ill-equipped to respond [7].

Guidelines designed to assist mainstream media outlets to report on suicide in a sensitive manner are widespread and have led to improved safety and quality of communication about suicide [8]. They also appear to be linked to a reduction in suicide rates [9]. However, until recently, no guidelines existed that targeted either social media or young people, who are now creators of their own content.

In response to this, we developed the #chatsafe guidelines [10], which were the first set of guidelines in the world specifically designed to better equip young people to communicate safely on social media about suicide. They were developed using the Delphi consensus method in partnership with young people, media, and suicide prevention professionals [11]. The guidelines consist of five sections: (1) things to consider before you post about suicide; (2) sharing your own thoughts, feelings, or experience with suicidal behavior; (3) communicating about someone you know who is affected by suicidal thoughts, feelings, or behaviors; (4) responding to someone who may be suicidal; and (5) memorial websites, pages, and closed groups.

The guidelines were supported by a national social media campaign designed to make the content of the guidelines more accessible to young people. The campaign was co-designed with young people from across Australia [12] and was initially rolled out nationally in 2019, reaching around 1.5 million young people. Together, the guidelines and campaign make up the #chatsafe intervention.

### Aims and Hypotheses

The overall aim of the study is to assess the impact of the #chatsafe social media intervention on young people’s safety and confidence when communicating on the internet about suicide. In this study, the treatment group who will receive the #chatsafe social media intervention will be compared to the control group who will receive social media content providing education on sexual health and well-being.

It is hypothesized that (1) At postintervention (T2), the treatment group will have higher mean scores than the control group on their understanding and compliance with the #chatsafe guidelines, as measured by the #chatsafe online safety questionnaire. Specifically, this will be assessed by improvements in their understanding of language to use or not use, support services available to them, how to share their own experiences of suicide safely, how to communicate about those who have died by suicide, and how to support someone that they are worried about. (2) At the postintervention time point (T2), the treatment group will have higher mean scores than the control group on self-reported (i) safety and confidence when communicating on the internet about suicide, (ii) willingness to intervene against suicide on the internet, and (iii) perceived internet self-efficacy. (3) It will be safe and acceptable to share the #chatsafe intervention with young people entirely via social media (assessed weekly throughout the intervention and postintervention [T2]). (4) Knowledge gains made in the intervention group at T2 will be maintained at T3.

Subgroup differences in perceptions toward the intervention and outcomes of the intervention (ie, gender, sexuality, age groups, and level of social media usage) will also be examined.

### Pilot Data

The #chatsafe intervention was tested in a pilot study in 2019-2020 [13]. The aims were to assess the acceptability, safety, and feasibility of the intervention as well as its impact on perceived self-efficacy, willingness to intervene against suicide on the internet, and safety of web-based communication about suicide. The study adopted a single-group pre- and posttest design. Participants were 189 young people aged 16-25 years recruited from 3 states in Australia via social media advertising. The intervention comprised 3 #chatsafe posts each week on the #chatsafe Instagram page. Participants were sent 1 post via direct message each week, for a period of 12 weeks. The results indicated that participants found the intervention acceptable. Participants also demonstrated improvements in perceived ability (*P*<.001) and intent to intervene against suicide (*P*<.001), and in several domains of internet self-efficacy, for example, problem-solving on the internet (*P*<.001). They also demonstrated improved web-based behavior, including monitoring their social media posts and responding directly to someone who may be suicidal.

Although these findings painted a promising picture, the pilot study was limited by the lack of control group. Therefore, the aim of this study is to test the efficacy of the #chatsafe intervention in a randomized controlled trial (RCT).

## Methods

### Study Design

The study uses a pragmatic, parallel, superiority randomized controlled design, where the intervention condition (the #chatsafe suicide prevention intervention) is compared to a control condition (sexual health content). It will be conducted over an 18-month period with up to a 12-month recruitment period and an 8-week intervention phase.

Participants will be assessed via self-report surveys at 3 time points: time 1 (T1)—baseline; time 2 (T2)—8-week postintervention commencement; and time 3 (T3)—4-week postintervention. In addition, participants will be asked to complete a short weekly survey to monitor safety and allow evaluation of each piece of social media content. All participants will be reimbursed for their time at a rate of Aus $45 (US $31.95) per hour.

**Figure 1:**
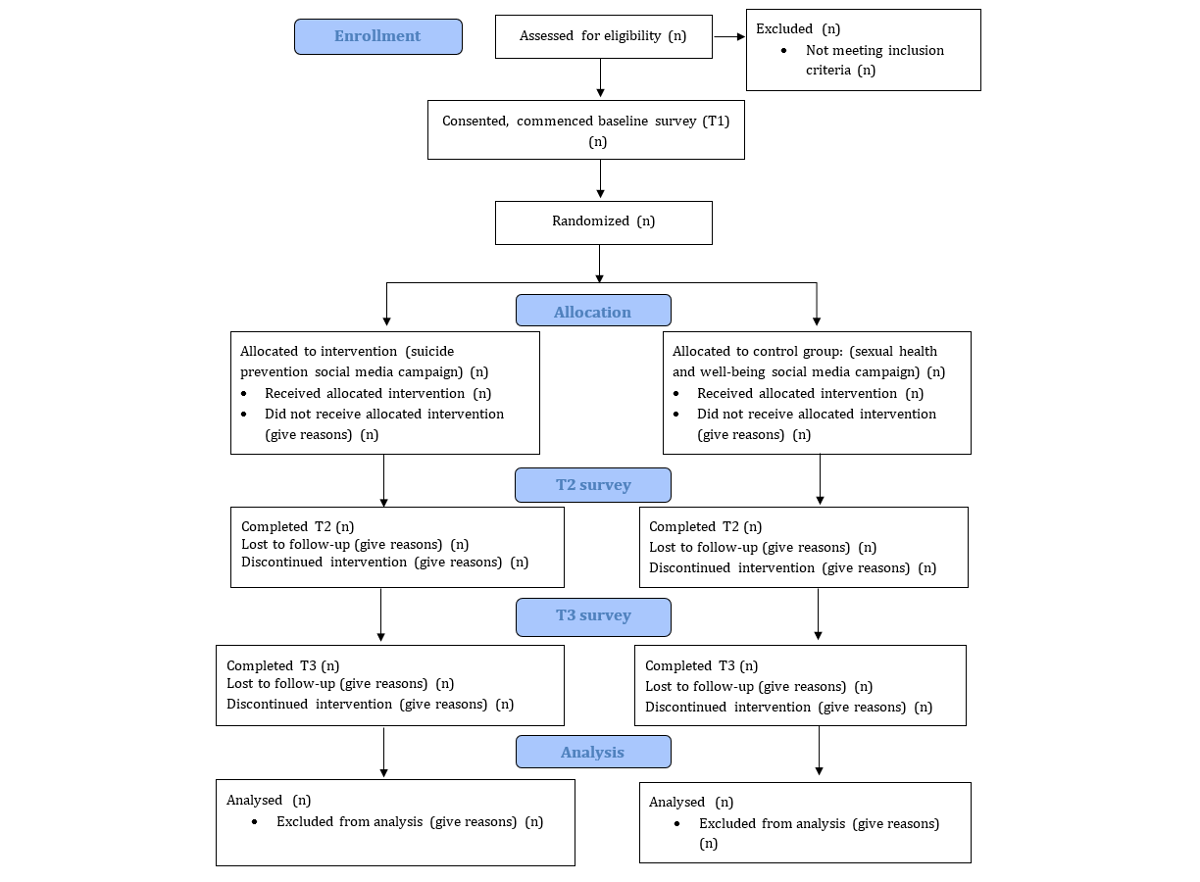
Consolidated Standards of Reporting Trials flow diagram of participants for enrollment, allocation, T1, T2, T3, and analysis stages.

The study is being led by researchers based at Orygen and the Centre for Youth Mental Health, University of Melbourne, Melbourne, Victoria, Australia. It will be conducted and reported in accordance with the Consolidated Standards of Reporting Trials statement [14] (Figure 1).

### Participants and Recruitment

Participants will be 400 young people (200 per arm of the trial). Inclusion criteria are being aged between 16 and 25 years, living in Australia, having used social media to communicate about suicide or self-harm *or* having seen suicide- or self-harm–related information on social media, having not previously participated in a research study that was focused on evaluating suicide prevention or sexual health–related content on social media, considering oneself as being an active social media user, and being willing to share one’s mobile phone number with the research team so that one can be contacted with regard to trial-related communications.

Participants will be recruited via social media advertising using Orygen’s social media accounts. Young people who wish to participate will be directed to a brief self-assessment questionnaire to assess eligibility. They will then automatically be directed to a web-based consent form, which explains the key aspects of study involvement and consent process. This is followed by the T1 questionnaire, after which, participants will be randomly allocated to either the intervention group (#chatsafe intervention) or the control group.

The participant flowchart is shown in Figure 1.

### Intervention

The intervention comprises an 8-week social media campaign. Intervention material will take the form of social media posts shared on public Instagram profiles created for the purpose of this study. As shown in Figure 1, the intervention group will receive the #chatsafe suicide prevention content, and the control group will receive the PROSPEct sexual health content. Both groups will receive 24 pieces of content; that is, 3 per week. All content will be delivered to participants’ mobile phones via SMS text messaging via REDCap (Research Electronic Data Capture), an electronic data capture tool hosted at The University of Melbourne. A description of the #chatsafe intervention (group A) is provided in Textbox 1. The content is based on the #chatsafe guidelines and was developed in partnership with young people; examples are provided in Multimedia Appendix 1.

The control content will comprise 8 weeks of sexual health content (PROSPEct content). A description of the control content (group B) is provided in Multimedia Appendix 2, and examples from both the treatment and control conditions are shown in Multimedia Appendix 1.

**The intervention content (group A, #chatsafe condition) by week.**

**Week 1: introduction**

- Co-design—how the campaign was developed in partnership with young people
- Campaign overview: introduction to campaign learning objectives
- What is safe and unsafe communication about suicide?

**Week 2: have you seen posts on the internet about suicide?**

- What is safe and unsafe communication about suicide? (continued)
- What to do if you’ve seen content that you think is unsafe
- What happens when you report content online?

**Week 3: sharing your own experiences of suicide on social media**

- Sharing experiences in a way that is helpful to you
- Sharing experiences in a way that is helpful to others
- Content warnings

**Week 4: asking someone if they are feeling suicidal**

- Myth busting—it is safe to ask about suicide
- Things to do before you approach someone
- Asking the question

**Week 5: navigating the conversation about suicide**

- If they say yes—What you can say to someone in suicidal distress?
- What to say and do if they say no
- How to exit the conversation and looking after yourself

**Week 6: when someone has died by suicide**

- Safely sharing the news online
- Checking in on your friends (and yourself)
- Memorializing the deceased or communicating after the death

**Week 7: helping a friend who has lost someone to suicide**

- Checking in with yourself
- Planning how to approach your friend
- Helpful things to say or do to help a friend affected by suicide

**Week 8: thank you and wrap up**

- Available resources and supports
- Co-design
- Thank you

### Study Outcomes

The primary outcome is safety when communicating online about suicide, as measured via the purpose-designed #chatsafe online safety questionnaire at T2. This measure was specifically designed for this purpose by 3 study authors (JR, LLS, and CC).

Secondary outcomes are (1) confidence when communicating on the internet about suicide, as measured via the purpose-designed #chatsafe online safety questionnaire at T2; (2) willingness to intervene against suicide on the internet, as measured via the Willingness to Intervene Questionnaire at T2 [15]; (3) internet self-efficacy, as measured by the Internet Self-Efficacy Questionnaire at T2 [16]; (4) safety of the weekly #chatsafe content, as measured by the purpose-built weekly evaluation and engagement survey; (5) acceptability of the #chatsafe intervention, as measured by the purpose-built T2 evaluation questions; and (6) safety of the #chatsafe social media intervention (as a whole), as measured by the number of (or absence of) adverse events recorded throughout the trial. Participant adverse events include (1) participant response to item 9 on the 9-item Patient Health Questionnaire [17] at baseline (time 1), time 2, or time 3. (2) Participant response to the 9-item weekly evaluation survey indicates distress, measured by participants selecting “very distressed” in response to the question “To what extent did you find the content this week distressing?” (3) Participant response to the T2 evaluation questionnaire indicates that a particular piece of campaign content made them feel distressed or at risk of suicide. (4) The participant directly contacts the research team via social media or email and reports distress or risk of harm to self. All adverse events will be responded to by the study team, in line with the study’s safety management strategy. (7) Feasibility of the #chatsafe social media intervention was measured by campaign reach via social media analytics, and participant retention or attrition via audit of study enrollment and withdrawal logs. (8) Self-reported evaluations of the acceptability of receiving the #chatsafe social media intervention were measured by purpose-designed study questions. Exploratory outcomes are as follows: (1) subgroup differences (gender, age, previous exposure to, previous experiences of suicide and self-harm, and level of social media usage) at T2 and (2) self-reported open-ended evaluations of the safety, feasibility, and acceptability of receiving the PROSPEct social media intervention for sexual health promotion in the control group at T2.

### Outcome Measures

Outcome measures together with the assessment schedule are presented in Table 1. Note that all participants complete the same questionnaires (T1, T2, T3, and purpose-designed 3-item weekly evaluation and engagement survey) regardless of the arm they are allocated to.

**Table 1:** Schedule of assessments and measures.

Measure	Baseline (T1, week 1)	Intervention (weeks 2-9)	Postintervention (T2, week 9)	Follow-up (T3, week 13)
Purpose designed #chatsafe online safety questionnaire	✓	N/A^a^	✓	✓
Patterns of Social Media Use Questionnaire (including 12 items to assess social media usage for the purposes of communicating about suicide and-self harm specifically)	✓	N/A	✓	✓
Perceived Internet-Self Efficacy Scale	✓	N/A	✓	✓
Willingness to Intervene Against Suicide Questionnaire	✓	N/A	✓	✓
Patient Health Questionnaire-9	✓	N/A	✓	✓
Purpose designed 7-items assessing exposure to, and personal experiences of, suicide and self-harm	✓	N/A	✓	✓
Purpose designed 9-item Weekly Evaluation and Engagement survey	N/A	✓	N/A	N/A
Purpose designed 1-item assessing participant reasons for withdrawal/noncomplete	✓	✓	✓	✓
Purpose designed 10-items assessing participants’ evaluations of the intervention content immediately postintervention	N/A	N/A	✓	N/A
Sexual Health Capacity Scale	✓	N/A	✓	✓
Self-Efficacy Protective Sexual Behaviours Scale	✓	N/A	✓	✓
Sexual Health Questionnaire	✓	N/A	✓	✓

^a^N/A: not applicable.

### Randomization and Treatment Allocation

Participants will be randomized to the intervention or the control group using block randomization with varying block sizes. The allocation ratio will be 1:1. The randomization schedule is computer generated by independent information technology personnel with guidance from the study statistician (MS). The schedule will then be implemented by a member of the study team (other than the statistician) into the REDCap database management software for allocating treatments to individual participants. Except for the study statistician, the study team members are not blinded to the treatment allocations. The study statistician will not have access to the randomization component of the database and will not be informed about the treatment allocations and will therefore remain blinded. As the study statistician will not be involved in any aspect of this study's operations other than data analysis, there are no circumstances we could foresee under which the study statistician would be required to be unblinded.

### Statistical Analysis

At baseline, state of residence, postcode, age, Aboriginal or Torres Strait Island status, gender identity, sexual orientation, primary language spoken at home, cultural background, and educational and occupational background will be reported using descriptive statistics and will be checked for imbalance between trial arms. The moderating effect of gender (male, female, trans, and gender diverse), age group (ie, 16-20 and 21-25 years), time spent on social media, psychological distress, and previous experience of suicide and self-harm will be considered in analyses as covariates.

Analyses will be performed on an intention-to-treat basis, where all individuals randomized will be included in the analysis by their allocated trial arm status regardless of whether they received all, part, or none of the intended treatments. For the primary analysis, we will use linear regression to estimate the difference in the mean changes between the intervention and control arms at T2 in the outcome safety when communicating online about suicide, as measured via the #chatsafe online safety questionnaire. This analysis will adjust for T1 scores. Multiple imputation will be used to address attrition bias, with 50 imputation samples generated using chained equations. We will conduct 2 sensitivity analyses. One sensitivity analysis will be undertaken using complete cases only (ie, repeating the primary analysis but only analyzing participants who have complete T1 and T2 data). The second sensitivity analysis will use multiple imputation and include the following potential moderating factors as covariates: gender (male, female, transgender, and gender diverse), age group (ie, 16-20 and 21-25 years), time spent on social media, psychological distress, and previous experience of suicide and self-harm.

The secondary outcomes at T2 and T3 will be analyzed based on the primary analysis and the 2 sensitivity analyses. We will use linear regression for continuous outcomes, logistic regression for binary outcomes, and negative binomial regression for counts.

Potential iatrogenic effects will be analyzed on a weekly basis and will serve as an interim analysis throughout the course of the study. If the weekly content is assessed as “very distressing” by more than 20% of participants per week, the safety monitoring committee (SMC) will be consulted, and the content may be withdrawn. The acceptability of each week’s content will be analyzed and reported as descriptive statistics (frequencies and percentages).

### Sample Size and Statistical Power

This is the first study to investigate the #chatsafe intervention in an RCT and the first to use the proposed measures. As such, there are no previous data to guide the possible effect of the intervention, and therefore the sample size is required; however, a sample of ~400 participants (200 per arm) should be sufficient to assess the primary study outcome with a 5% significance level and 80% power.

The pilot study [13] recruited a total of 507 participants within a 3-month period, resulting in a final sample of 189 young people who completed all 3 time points. Note that the intervention period for this study was 12 weeks compared to 8 weeks in this study. As such, data from the pilot study on rates of recruitment and attrition indicate that the recruitment period of up to 12 months will be more than sufficient to achieve the required sample size.

In an effort to reduce attrition, all participants will be sent a SMS text message by a research team member halfway through the intervention period (week 4), as well as at T2 and T3 if they have not completed their survey 4 days after being sent their survey. This will encourage participants to complete their survey if they have not already done so and will provide them the opportunity to raise any concerns or questions.

### Ethical Considerations

The study has been approved by the University of Melbourne Human Research and Ethics Committee (ID 2022-24238-32907-3). All participants will be required to provide written consent prior to commencement of the study.

The study will be carried out in accordance with the principles contained in the Declaration of Helsinki and the Australian National Health and Medical Research Council (NHMRC) National Statement on Ethical Conduct in Human Research and the NHMRC Australian Code for the Conduct of Research. In addition to being assessed at T2, safety will also be tracked using the weekly evaluation and engagement survey to identify any distress that is attributed to the intervention (see Table 1). Previous distress and suicide risk will be deemed as that reported during the baseline assessment. Further relevant risk will not be reported unless adverse events are reported by the participant or other study team members, which are deemed to possibly having been caused by study activities such as distress because of the intervention or because of interaction with the study team.

As per the pilot study, an SMC has been established. The main purpose of the committee is to oversee the safety of the trial. It comprises the study Chief Investigator (JR), members of the study team, as well as an Orygen Sponsor Operations representative, a statistician, a clinician, and a subject-matter expert. An initial meeting will be held prior to the commencement of recruitment to review the study risk assessment and the determination of safety oversight procedure. The research team will meet fortnightly for the duration of the trial, and a standing function of these meetings will be to review safety incidents and refer any relevant matters to SMC. All members of the SMC will be convened on a study-specific basis (ie, any time a serious safety concern is raised). The SMC will make recommendations as to appropriate risk assessment and mitigation measures, including but not limited to decisions as to whether the study should be amended or put on hold.

Finally, detailed safety protocols have been established and overseen by the trial psychologist (SR) and Orygen’s Sponsor Operations team (Multimedia Appendix 3).

### Youth Participation

Young people have been involved in the preparation of this study in multiple ways. First, the entire #chatsafe program of work has been conceptualized, designed, and conducted in partnership with young people. The #chatsafe guidelines were created using the Delphi consensus method, with young people as an expert panel [11]. The first #chatsafe social media campaign was cocreated in partnership with over 140 young people [12].

The study was presented to Orygen’s Youth Research Council in November 2021, and feedback on intervention development and study conduct was obtained. A total of 3 co-design (N=46) and 1 user testing (N=10) workshops were conducted in April and May 2022 to help create the trial content. A total of 3 youth advisors helped to develop the participant information consent form and the #chatsafe online safety questionnaire. Finally, we are currently recruiting 2 paid youth advisors to this study to assist with participant recruitment and engagement. At the end of the study, they will assist with the interpretation and dissemination of the study findings, including conference presentations and journal articles.

## Results

The funding for this project was awarded in November 2020 by the Australian Research Council; however, progress was delayed down due to the impacts of the COVID-19 pandemic. Approval from the University of Melbourne Human Research and Ethics Committee was provided in October 2022. Recruitment into the trial began in November 2022 and study completion is anticipated by June 2024. The trial is registered with the Australian and New Zealand Clinical Trials Registry (ACTRN12622001397707).

## Discussion

This will be the first RCT internationally to test the impact of a social media intervention designed to better equip young people to communicate safely on the internet about suicide.

Social media platforms are commonly used by young people to communicate about suicide. While users rarely set out to cause harm to themselves or others, the potential for harm exists [18,19]. Therefore, it is essential that young people are adequately equipped to safely communicate on the internet about suicide. Guidelines regarding safe reporting of suicide exist for mainstream media [20]; however, until recently, no such guidelines have been developed by, and for, young people. Also, existing media guidelines do not specifically address the challenges and opportunities offered by social media. Although media campaigns are gaining traction in the suicide prevention sector, to date, they have not been developed with young people in mind nor have their impact on behavior change, in particular among young people, been tested.

The development of the #chatsafe intervention not only addresses these gaps, but it also represents a paradigm shift. It shifts the narrative away from advising young people to avoid engaging in suicide-related communication on social media—a strategy that is clearly not working—toward an approach that seeks to better equip young people to communicate safely. The guidelines themselves have already had significant traction not just in Australia but also internationally. They have been adapted for 11 additional countries and downloaded around 120,000 times [21]. In addition to being freely available on the Orygen website, they are also available via both the Facebook and Instagram Safety Centers, thereby significantly extending their reach [22,23]. The social media content has been widely disseminated via platforms such as Instagram, Facebook, Snapchat, and YouTube. There are few suicide prevention interventions that have the potential to be delivered at scale in this way.

This study proposes a rigorous and novel methodology. Until now, research into suicide and social media has been largely cross-sectional in nature and has examined the association between social media use and suicidal thinking. No studies have tested suicide prevention interventions delivered via social media using robust research designs. This study will build on a previous pilot study (La Sala et al, unpublished data) and will provide a robust evidence base for this work.

This work is timely. Given the rising rates of youth suicide in Australia and the acceptability of social media among young people, incorporating social media–based interventions into the suicide prevention landscape is an obvious next step. This intervention, if effective, could also be extended to international settings, thereby improving online safety for young people not just in Australia but globally.

## Appendix

Multimedia Appendix 1 Intervention content examples.

Multimedia Appendix 2 The control content.

Multimedia Appendix 3 Overview of safety management strategy.

Multimedia Appendix 4 Peer review reports from the Discovery Projects 2021-2023 - Australian Research Council (Canberra, Australia).

## Abbreviations

NHMRC: Australian National Health and Medical Research Council
RCT: randomized controlled trial
REDCap: Research Electronic Data Capture
SMC: safety monitoring committee

## Data Availability

All data analyzed during this study will be included in any published articles (and their supplementary information files). The participants of this study did not give written consent for their data to be shared publicly, so due to the sensitive nature of this research, supporting data are not available.
