# Supplementary material for: Testing the Impact of the #chatsafe Intervention on Young People’s Ability to Communicate Safely About Suicide on Social Media: Protocol for a Randomized Controlled Trial
Source: JMIR Res Protoc. 2023 Feb 17;12:e44300. doi: 10.2196/44300 (PMC9984994; doi:10.2196/44300)
Supplement: Multimedia Appendix 1 [file resprot_v12i1e44300_app1.docx]

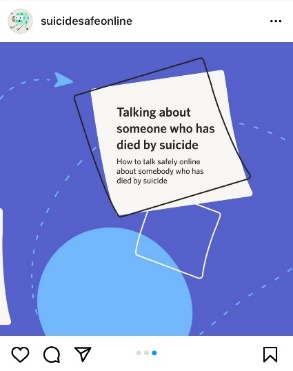

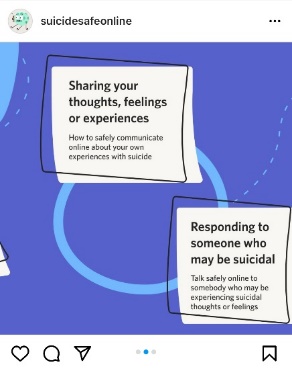

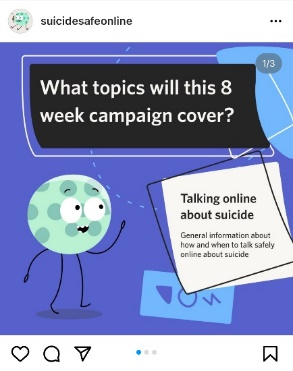
Examples of #chatsafe campaign content

**Accompanying caption:**

Suicidal thoughts in young people are more common than many people realise. Although many young people don’t ever act on these thoughts, some do. For many, social media can be a safe and non-judgmental place to share what’s going on for them or to reach out for support.

Because of this, you might have already come across suicide-related content online, such as images, videos, personal stories, pop-culture references or stories about celebrity suicides. Some people will go looking for information or help for themselves, or they might be wanting to support their mates. It's really important to upskill everyone in our community so that they have the tools on how to safely share and/or respond to suicide-related content online, in ways that promote online safety for themselves and others.

Our research has shown that it is possible to upskill young people to safely community about suicide on social media. By equipping young people with evidence-based information and skills, we can create safer online communities and help prevent future youth suicide.


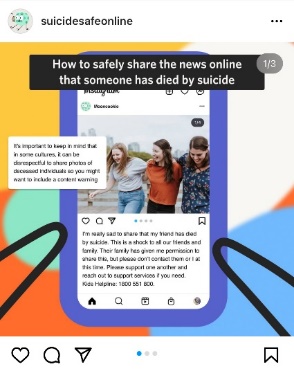

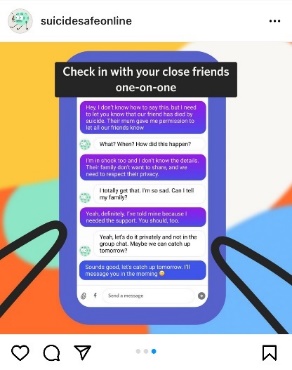

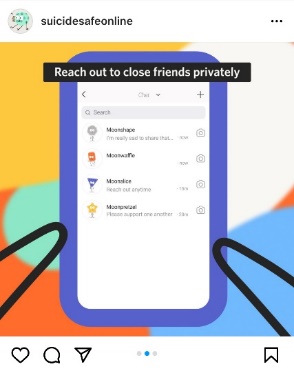


**Accompanying caption:**

It’s difficult to describe how tough it is to lose someone in your community to suicide. Given how hard suicide can be to talk about, many will turn to online spaces to communicate about what’s happened.

This might look like creating posts to remember the person and share the news with their online community. In these times, it’s super important to keep our safe communication tips in mind.

If you want to safely share the news online that someone has died by suicide, make sure to:

1) Verify your facts (has this person’s death been confirmed by a reliable source, such as a trustworthy news source or the family?
2) Ask the person’s family or next of kin for permission to share the news online;
3) Message people privately; and
4) Do not share details of how, when, and where the person died.

Together, these actions can help to protect other people experiencing suicidal thoughts and feelings themselves who might see the post.

Examples of PROSPEct campaign content


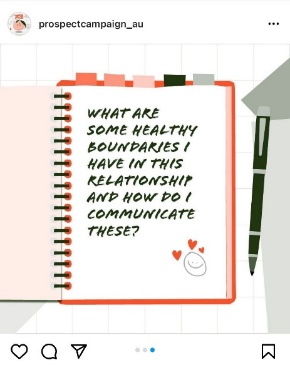

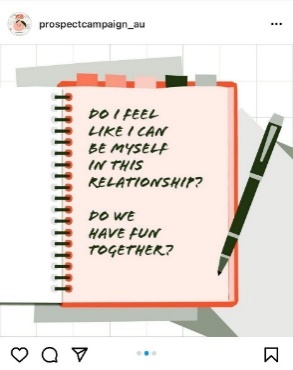

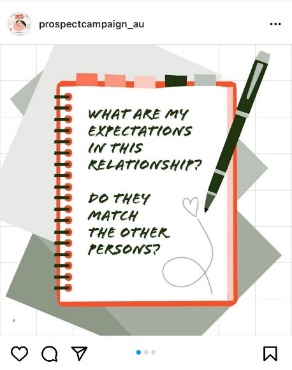


**Accompanying caption:**

Healthy relationships often look different for people – we all have different needs and desires after all. Noticing unhealthy behaviours (like manipulation, controlling your actions, isolating you from friends) and & striving for healthy behaviours (like mutual respect, support, trust) should be a big priority in any relationship.

Remember that relationships can be friendships, the relationship you have with your family, romantic, sexual, casual or serious. Check out our journal prompts above and ask yourself these questions about a relationship you’re in!


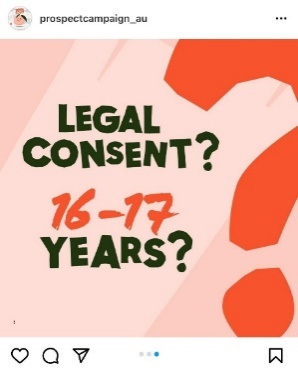

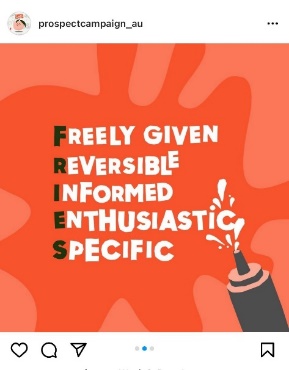

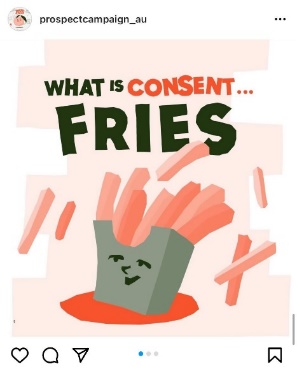


**Accompanying caption:**

When we hear ‘no means no’, we want to shrivel up. We need to look for more than just the absence of a ‘no’, but the presence of an affirmative yes!

The legal ages varies between 16-17 years old in Australia depending on the state you’re in. There are also times that legal consent can’t ever be given or received that you need to know about:
• If somebody is under 12 years old
• If somebody is 12-15 years old, sex is not legal if one person is 2 years older than the other
• If somebody has a position of care/authority (e.g. teacher), they cannot have sex with somebody aged 16-17 in their care

Don’t forget, if somebody is too high/drunk to get behind the wheel, they are also too intoxicated to give/gain consent. If in doubt, just wait until another time when you can be sure you’re both truly willing!
