# Supplementary material for: Testing the Impact of the #chatsafe Intervention on Young People’s Ability to Communicate Safely About Suicide on Social Media: Protocol for a Randomized Controlled Trial
Source: JMIR Res Protoc. 2023 Feb 17;12:e44300. doi: 10.2196/44300 (PMC9984994; doi:10.2196/44300)
Supplement: Multimedia Appendix 2 [file resprot_v12i1e44300_app2.docx]

| The intervention content (Group B, PROSPECT condition) | | |
| --- | --- | --- |
| Week | Weekly content theme | Specific content theme |
| 1 | Introduction and setting the tone | 1.1 What is PROSPECT? |
|  |  | 1.2 Engaging with sexual health content |
|  |  | 1.3 Co-Design |
| 2 | Understanding our bodies | 2.1 A rundown of body parts |
|  |  | 2.2 The menstrual cycle |
|  |  | 2.3 Body image |
| 3 | Widening the concept of what sex and consent are | 3.1 Understanding what informed consent is |
|  |  | 3.2 Gaining/giving informed & affirmative consent |
|  |  | 3.3 Exploring what sex is |
| 4 | STI awareness | 4.1 What are STIs and symptoms |
|  |  | 4.2 Using barrier methods |
|  |  | 4.3 Getting STI tests |
| 5 | Contraception options | 5.1 Contraception options 1 |
|  |  | 5.2 Contraception options 2 |
|  |  | 5.3 Emergency contraception |
| 6 | Diversity and Pleasure | 6.1 Gender diversity |
|  |  | 6.2 Attraction/Orientation |
|  |  | 6.3 Pleasure |
| 7 | Relationships | 7.1 Online relationships and activity |
|  |  | 7.2 Healthy vs unhealthy |
|  |  | 7.3 Healthy communication and boundaries |
| 8 | Further support and thank you | 8.1 Sexual health awareness & check ups |
|  |  | 8.2 Finding youth friendly services |
|  |  | 8.3 Thank you |
