# Supplementary material for: Testing the Impact of the #chatsafe Intervention on Young People’s Ability to Communicate Safely About Suicide on Social Media: Protocol for a Randomized Controlled Trial
Source: JMIR Res Protoc. 2023 Feb 17;12:e44300. doi: 10.2196/44300 (PMC9984994; doi:10.2196/44300)
Supplement: Multimedia Appendix 3 [file resprot_v12i1e44300_app3.docx]

## Overview of safety management strategy

| **Examples of incidents whereby individuals may report distress and/or risk of harm that requires follow up:** |
| --- |
| 1. Participant withdrawal from the research study 2. Participant response to item 9 on the PHQ-9 survey at baseline (Time 1), Time 2 or Time 3 3. Participant response to the 9-item weekly evaluation survey indicates distress. 4. Participant response to T2 evaluation questionnaire indicates that a particular piece of campaign content made them feel distressed or at risk of suicide. 5. Participant directly contacts the research team via social media or email, and reports distress or risk of harm to self. 6. Participant does not participate in the study for a period of two-weeks. 7. Member of the public (non-participant) responds to social media campaign via direct message on any social media platform, and reports distress or risk of harm to self. |

The safety response activated will differ in each of the aforementioned circumstances. Each of these safety responses are outlined in below according to the preceding event where risk is identified.

These safety incidents and responses have been guided by two pilot studies previously conducted by the research team.

| **Incident** | **Details** | **Safety Escalation Plan** |
| --- | --- | --- |
| **1. Participant withdrawal from the study** | Participant contacts the research team to withdraw from the study or selects “I wish to withdraw from the study” at any time point. | Following their request to withdraw from the study, participants will be provided with the option of having the study psychologist contact them. If participant responds with their contact details and request follow up from the study psychologist: we will then pass on their details to the study psychologist, either Simon Rice or Eleanor Brown, and they will contact the participant to conduct a risk assessment.   1. If the participant is at immediate risk: emergency services will be contacted and a staff member will remain in contact with them until help arrives. Any concerns or subsequent referrals will be clearly documented and reviewed during a SMC meeting which will be called immediately. 2. If the participant is not at immediate risk: the study psychologist will speak with the participant to ensure that they are aware of what support services are available to them and how best to access those.   If the participant responds and declines to be contacted by the study team: the study team, with the support of the study psychologist, need to assess whether they are still concerned about the young person. If the study team are still concerned, then they will take another possible step to re-contact the participant after appropriate time period to “check in”. Other steps may include: contacting them via email, contacting them via a social media platform (if we have details), or any other information that the study team have at hand. |
| **2. Participant response to item 9 on the PHQ-9 survey at Time 1, Time 2 or Time 3** | Participants will be asked to complete the 9-item Patient Health Questionnaire (PHQ-9) at Time 1, Time 2, and Time 3. Item 9 asks participants, “over the last two weeks, how often have you had thoughts that you would be better off dead, or of hurting yourself”. Participants can respond on a 5-point Likert scale ranging from ‘not at all’ to ‘nearly every day’.  This automatic escalation response will be activated any time a participant selects “several days”, “more than half the days” or “nearly every day” to item 9. | In this instance, participants will receive a pre-programmed message within the survey (hosted by RedCap). This message will advise them to consider whether participating in this study is within their best interest at this time, remind them that involvement in this study may involve exposure to, and questions about, suicide-related content, and encourage them to seek support if they need it.  Participants will have the option of selecting:   1. *I would like to continue participating in this study* 2. *I would like to stop my involvement in this study.*   Participants who select option 1 continue as normal.  Participants who select option 2 will follow the process outlined in **Incident 1.** |
| **3. Participant response to the 9-item weekly evaluation survey indicates distress** | Participants will receive a short 3-item evaluation and engagement survey with the campaign content each week. These three items ask participants how the content made them feel, how helpful they found the content, and whether or not they would share the content with others.  An automated response will be activated if a participant indicates potential iatrogenic effects following exposure to the campaign content (i.e., selects ‘Very distressed’ in response to the question about how the content made them feel).  In this case, they will receive a message advising them to contact a helpline, their GP, headspace or other support services. Participants will also be reminded that they can snooze the campaign content for one week or withdraw from the study altogether. Any distress or choices to opt out of the study will be documented in the adverse reactions log. | Participants will be provided with contact details for available support services and will also be invited to consider snoozing the campaign content for one week, or withdrawing from the content altogether.    Participants who select that they would like to withdraw follow steps outlined in **Incident 1.**  Participants who select that they would like to snooze the campaign content for one week, have a one-week study pause.  Participants who select that they would like to continue, proceed as normal. |
| **4. Participant response to T2 evaluation questionnaire indicates that a particular piece of campaign content made them feel distressed or at risk of suicide** | An automated risk management response will be activated at T2 if a participant indicates that a particular piece (or multiple pieces) made them feel suicidal during the course of the campaign. The question will ask participants “At any point during the intervention, did the campaign content you viewed as part of the study, cause you to feel suicidal, unsafe or cause you to experience the urge to self-harm?’. | Participants who respond “yes” to this question will receive the same messages from **Incident 2.** If the participant wishes to withdraw from the study, the process from **Incident 1** will be followed.  Participants who wish to continue will then be given the option to detail what it was about the content they found distressing. They will also be provided with contact details for professional support services, the study email address to which participants are able to receive practical study support from the research team during business hours. |
| **5. Participant directly contacts the research team via social media or email, and reports distress or risk of harm to self** | If a participant contacts the research team in any way (e.g. email, social media message, social media comment), the research team will respond in a way that is appropriate to that particular message.  Participants will always be directed to: eheadspace 1800 650 890 https://www.eheadspace.org.au/, or Kids Helpline 1800 55 1800 https://kidshelpline.com.au/ Participants will also be reminded that they can withdraw from the study or snooze the campaign content for one week. Any distress or choices to opt out of the study will be documented in the adverse reactions log. | The study psychologists and Safety Monitoring Committee (SMC) will be called upon any time contact from participants indicates that exposure to the campaign content or participation in the research study has caused distress. The most appropriate response and follow up will be decided by the SMC.  In the instance that the participant wishes to withdraw from the study, the process from **Incident 1** will be followed. |
| **6. Participant does not participate in the study for a period of two-weeks** | Participants are asked to complete the Weekly Evaluation and Engagement Survey each time they receive a new piece of content (Weeks 1 – Weeks 8).  If participants do not respond for a period of two weeks or more, this incident plan will be activated. | If a participant does not respond to the weekly content for a period of 2-weeks or more, participants will receive an SMS checking in with respect to their engagement and wellbeing. They will be reminded of their option to elect to withdraw from the study at any time.  If the participant wishes to withdraw, the process from **Incident 1** will be followed. |
| **7. Member of the public (non-participant) responds to social media campaign via direct message on any social media platform, and reports distress or risk of harm to self** | As the social media campaign content will be hosted on Instagram, anyone is able to view and respond via direct message to the research team. For safety reasons, the comment function on the study social media pages will be turned off. However, members of the public will be able to send private messages to the social media account, and these messages will be monitored daily by the research team. | If someone indicates distress through social media, the Communications team at Orygen will respond directly in accordance with Orygen social media procedures.  The research team will attempt to find out some information about that individual (if available, i.e. location, any other recent public posts that may identify the person, etc.) If appropriate, the message/post will also be reported to the platform’s safety centre. All steps taken will be documented in the adverse reactions log. |
