# Supplementary material for: Testing the Impact of the #chatsafe Intervention on Young People’s Ability to Communicate Safely About Suicide on Social Media: Protocol for a Randomized Controlled Trial
Source: JMIR Res Protoc. 2023 Feb 17;12:e44300. doi: 10.2196/44300 (PMC9984994; doi:10.2196/44300)
Supplement: Multimedia Appendix 4 [file resprot_v12i1e44300_app4.pdf]

Detailed Assessments

Assessor Id	Actions
B	Hide

Investigator(s)/Capability
A very strong and appropriately diverse team of investigators. The academic investigators have a strong track record in suicide prevention and young people, with a staggerring amount of successsfully completed funded research from a range of sources, both industry and government. Appropriately incuded is an Aboriginal academic as a CI as well as the casual employment of young people as youth advisors. They also have the support of appropriate industry partners, such as facebook and strong links with international collaboratiors.
Project Quality and Innovation
The project asks to trial and evaluate an intervention created by the team (chatsafe). This educative 12 week online programme aims to positively influence the ways in whch young people engage with suicide online. The methodology is detailed and appropriate and consists of completion of the program by young people, followed by an analysis of their online discussion over the following 12 weeks with a comparison of their online presence compared to the 4 weeks prior to completing the programme. The team note that this is an innovative approach to an evaluation, occurring in real time. However, it is not clear that a Discovery application is the most appropriate source of funding for this research project, based as it is on an evaluation (however novel) of a product created by the same team. It might be that such a project is better suited to a linkage application or a commercial arrangment with an organisation such as Facebook. It is also not clear if the programme should be evaluated by the team who created it. It would be good to know how this potential conflict might be managed.
Benefit
There is value in creating and evaluating interventions which work to educate young people in suicide prevention, and this project is clearly well formulated, supported by a strong team and innovative in its research design. Online safety for young people and youth suicide prevention are both a focus of the Federal Government with an increasing youth suicide rate of vital imprtance to the community. This project has the potential to contribute to educating young people to manage their communications about suicide
Feasibility
The team is successful in managing large externally funded research projects. The project is well designed and each member of the team bring an appropriate skill set to the project. I have no doubt this prokect will be successfully completed

Assessor Id	Actions
C	Hide

Investigator(s)/Capability
This is a strong team with specialist expertise in suicide prevention, social media, computer science, broader prevention science, policy impact and industry 'know-how', as well as track record in working collaboratively, including (at least for some members) on the development and piloting of the #chatsafe initiative. Having a team of this calibre is especially important for a project of this nature as such research is ethically and methodologically very challenging. CI Robinson is well positioned to be leading this project given her role as Head of the Suicide Prevention Research Unit at Orygen. She is highly regarded in the mental health and suicide prevention space in Australia, bringing considerable expertise and an established network of collaborations, as well as the opportunity to oversee and lift the profile of the project through her substantive role. CI Pirkis is similarly very experienced with specialist expertise in suicide prevention research that is foundational to the proposed project. CI Krysinska's experience as a research psychologist should help to monitor and safeguard the wellbeing of participants. CI Rice's experience with the development of the #chatsafe guidelines will be valuable as will CI La Sala's with project management, given her lead role in the pilot of the #chatsafe study. The expertise of international partners PI Kautz and PI Silenzio will make an important scientific contribution to the study. The inclusion of PI Finlay's experience in Aboriginal and Torres Strait Islander public health will be critical as will her background in #chatsafe as a consultant with Orygen. PI Skehan's experience in suicide prevention work, her knowledge exchange interests and expertise, as well as policy and practice reach, will benefit the program as will PI Goodrich's role as an industry partner with considerable experience in digital innovation. While Facebook is not partnering on the research, Garlick's involvement in an advisory capacity will be critical to end-user translation, as will her working understanding of #chatsafe through her current association with Robinson and Orygen. It is also great to see the involvement of youth advisors. While details on their role are sparse (and hence I'm not convinced quite meet the claim of 'participatory design'), a project of this kind will be considerably enhanced by efforts to involve them at every stage in a timely and meaningful way. Overall, excellent researcher capability.
Project Quality and Innovation
This project has a clearly evaluative intent in using #chatsafe campaign content and network and linguistic analysis in investigating the impact of using social media platforms to improve online safety for young people when communicating about suicide. In this way, it addresses a critical aim while working productively with tensions between perspectives suggesting social media may contribute to suicide and potentially prevent it. In evaluating the #chatsafe intervention, the project is simultaneously ambitious in its intent (a 'first') and rigorous in its approach while endeavouring to mitigate risk. That said, I'm unsure that the applicants claim 'the study represents a paradigm shift' and 'proposes a novel methodology' is well argued beyond research into suicide being largely epidemiological to date with few studies testing suicide prevention interventions delivered via social media. This is certainly a gap the research as detailed will fill, but whether linguistic and network analysis are still considered cutting edge or novel as methodologies, may be slightly questionable. The applicants do make the point, though, that these methods have not been previously used to evaluate a social media-based suicide prevention intervention for young people, and that is certainly important to note. My initial concerns that recruitment of the numbers indicated might be too ambitious are offset by the figures provided in successful recruitment and retention for the pilot. Reference is made to screening, eligibility, consent etc. but little detail is provided on this. Notwithstanding limited space, more detail on the ethical aspects of the study would have been useful. Reference is made to the #chatsafe intervention being co-designed in partnership with young people, along with a commitment to continuing a 'participatory' approach throughout this study, but little detail is provided on this – something that I think would further distinguish the proposed study and underline its significance.
Benefit
The case for benefit is evident and well argued. Suicide remains a significant and persistent social problem with far-reaching impacts, given it is the leading cause of death for young Australians. The role of social media is recognised but not well understood – nor has there been any intervention like #chatsafe that has been rigorously evaluated in terms of reducing risk. The benefits to young people in learning how to communicate safely could be far-reaching as could the ways in which the findings might benefit the social media industry as they endeavour to operate platforms safely. The timeliness for government policy has been well argued. What has been missing has been sound evidence-based advice and this project appears very well planned and positioned to generate this.
Feasibility
The proposed study is very strong on feasibility, with #chatsafe having previously been tested with young people in several states of Australia. This suggests that some of the very challenging issues associated with a study of this kind have been identified and mitigated against, especially in regard to safety with content, methods and processes. The technological and analytical logistics alone are considerable over the proposed period of the project but the methods identified to deliver the
Intervention appear to be well worked through. Given a very realistic budget and considerable in-kind contributions, as well as work being undertaken through substantive roles, there doesn't appear to be any major impediments to delivering on time and on budget. The fact that team members are very well networked, with a number of critical partnerships already established to support this project through recruitment, sharing campaign content and using the findings, will also contribute substantially to feasibility.

Assessor Id	Actions
A	Hide

Investigator(s)/Capability
This is an exemplary team. Lead CI Robinson is a leading national and international scholar in this field; and she is joined by a team of researchers also working in this field, and at the top of their game. The team is large and complex, involving researchers from two institutions in the United States as well as industry partners here in Australia. The project team as outlined looks to be comprehensive in covering off on mental health, social media, and youth suicide prevention expertise while also partnering with appropriate social media industry expertise and support to ensure the project design can be met. There is capacity for ECRs to be mentored by this senior team of key CIs -- the 0.8FTE CI is ECR and there is further capacity-building in the Senior Research Associate and Research Assistant teams. This is an excellent team that can clearly carry out the project as enunciated, and lead to implementation in practice, and at the policy level.
Project Quality and Innovation
This is a significant project operating within an important field to address a key social challenge -- rising rates of youth suicide. The innovation of the project lies in the testing of the #chatsafe online materials, and the methods used at different phases of the research process to deliver reliable findings. The engagement of linguists in the United States with whom some of the project team members have already worked offers interdisciplinarity and training opportunities to Australian ECRs. The clear tie-in of this project with existing policy considerations, and current guidelines discussions within social media giants such as Facebook, ensure both the quality and applicability of this project. The team is well-experienced to navigate the ethical difficulties of accessing young peoples' social media content which forms the core of the quality in this project.

This is a highly-applied project, and I would ask the researchers in the rejoinder to address the intellectual contribution of the project -- how will the project advance the field of youth suicide prevention and our conceptual framing of such research?
Benefit
I'm finding it difficult to write a lot in this assessment (!), as the case for quality, benefit, and the ability of the team to carry it out is clear to me. This project is of essential national benefit -- it is testing the efficacy of the #chatsafe materials as a way to understand and mitigate increasing rates of youth suicide. The research team has clear links into the national policy environment and so the evidence gathered in this project will undoubtedly feed into broader policy; and the research team's connections with Facebook (and by extension, its partner Instagram) will also ensure these guidelines are adopted by the social media platforms should this research show they are effective in assisting young people. The #chatsafe materials, therefore, and the research which is designed to test them, are of significant national and international benefit.
Feasibility
For all the reasons outlined above, this application has well-established the feasibility of the research. The recruitment methods of young participants have been piloted and shown to work; and the #chatsafe materials have also been piloted with this initial sample. The project team has the clear expertise to carry out the work; and the team is sufficiently large to ensure the project is completed on time and with significant research outputs. I imagine the project would still be feasible with slightly less administrative and research support (eg with both the RA and the SRA offering significant support); but that is a matter for the research leaders and the funding body. I strongly endorse the feasibility of both the project design and the team as outlined.

Rejoinder Status Submitted to ARC

Rejoinder Text

All assessors were extremely positive about the calibre of our team, the feasibility, quality and innovative design of the project, and the likelihood that it will have "significant national and international benefit" in terms of reducing youth suicide. The assessors asked for more information on:

1. Intellectual contribution and advancing the field: "Online safety … and youth suicide prevention are both a focus of the Federal Government [and] with an increasing youth suicide rate [are] of vital importance" (Assessor B). However, the role of social media in suicide prevention is poorly understood with little extant evidence on what constitutes safe and helpful online communication or the impact of social media interventions on suicide risk. This study will address these gaps thereby providing the "sound evidence base" that is currently absent (Assessor C). Additionally, it represents a conceptual shift advancing the field from typical studies examining the risks associated with social media to testing a novel intervention co-designed by young people. The project will be the first worldwide to employ a participatory framework and methods such as linguistic and network analysis to test a social media intervention in youth suicide prevention. The international partnerships will serve as a transformation model for future work, and our long-standing engagement with industry partners and policy makers will ensure "implementation in practice, and at the policy level" (Assessor A) - both urgently required.

2. Managing conflicts from internal evaluation: Gold standard in implementation science is for interventions to be first tested by those who create them, paving the way for future external replication. If studies are rigorously designed many benefits exist, e.g. greater understanding of the intervention and its aims. In this study our primary outcome will be assessed by the US-based PIs who were not involved in developing the intervention and will remain blind to time-point thereby minimizing risk of bias. In addition, open science practices will be adopted to increase replicability and transparency, including pre-registration of the trial, open data and code, and publishing the findings in an open access journal.

3. Relationship with Facebook: Assessor B asked if the project would be suited to a Linkage grant or commercial arrangement with Facebook; we disagree. Commercially Facebook's priorities do not align with ours and we agree with the other Assessors that the strength of the partnership with Facebook (and by association Instagram) lies in their capacity to facilitate sustainable end-user translation at scale, and our capacity to provide them with independent, evidence-based guidance.

4. Ethics: It was noted that as a team we are well equipped to manage the ethical aspects of the study (Assessor A) and we have published extensively on the ethics and safety of conducting suicide research (see CI Robinson's journal articles 1,7,9,11,30,56). Assessor C asked for more detail. In this study we will adopt the same standard ethics procedures as in the pilot e.g. ethical approval from The University of Melbourne; written informed consent; tried and tested risk protocols; and input from a study psychologist and an independent safety monitoring committee. All content has been co-designed and piloted with young people and all study protocols including consent forms, safety procedures etc. will be co-developed with, and reviewed by, young people. Participants will be reimbursed for their time. Well-being plans are used for all youth advisors and participants.

5. Youth participation: As recognised by Assessors B and C youth participation is central to this study. Assessor C suggested that more detail on this would "further distinguish it ... and underline its significance". Our team are at the forefront of developing participatory approaches in youth suicide research. In this study young people co-developed the guidelines and the campaign content to be tested (both now published). Paid youth advisors helped design the study and write the grant application. If funded, in addition to the 2 paid youth advisors, the #chatsafe youth network (n=161) will co-design and co-create any further campaign content. We have developed a partnership with RMIT and their design and communications students will co-develop the campaign strategy. Finally (as per the pilot) paid youth advisors will help with developing study protocols, participant recruitment and engagement, and in the later stages with data analysis, publication writing and co-presenting study findings.

We appreciate the assessors' description of our team as "exemplary" and their view that the project is "timely" and of "essential national benefit" given Australia's rising rates of youth suicide.
